# Endoscopic Surgical Repair of a Giant, Postoperative, Neglected Meningoencephalocele

**DOI:** 10.7759/cureus.6739

**Published:** 2020-01-22

**Authors:** Panagiota Kosmidou, Vasiliki Ntarladima, Antonios Katsimantas, Dimitrios Filippou, Christos Georgalas

**Affiliations:** 1 Otolaryngology - Head and Neck Surgery, Evangelismos General Hospital, Athens, GRC; 2 Urology, Mediterraneo Hospital, Glyfada, GRC; 3 Surgery, National and Kapodistrian University of Athens School of Medicine, Athens, GRC; 4 Surgery - Head and Neck, Nicosia University, Nicosia, CYP

**Keywords:** cerebrospinal fluid leak, meningoencephalocele, rhinorrhea, endoscopic treatment

## Abstract

Meningoencephalocele is a rare and potentially lethal disease, requiring early diagnosis and treatment. A 30-year-old male patient was diagnosed with a massive meningoencephalocele. His medical history included cerebrospinal fluid (CSF) rhinorrhea since the age of 7 years, which was attributed to right eye mining during infancy due to Coats disease. Following failed attempts of open surgical repair and CSF diversion during childhood, no further attempts of surgical management were made. He presented a long history of recurrent episodes of meningitis, resulting in long-lasting hospitalization in the intensive care unit. Eventually, he underwent surgical repair through an endoscopic multilayer approach. Subsequent endoscopic and radiological assessment demonstrated no recurrence during a follow-up period of one year. Endoscopic treatment is highly successful. An effective and definite surgical repair is of paramount importance in order to avoid life-threatening complications, improve patient’s and caregiver’s quality of life, and avoid unnecessary health-system costs.

## Introduction

Encephalocele or cranium bifidum is a neural tube defect characterized by the protrusion of brain tissue and overlying meninges through a bony deficit either along the midline of the cranial vault or at the base of the skull [1]. In the case that the meninges only protrude, the term meningocele is used [1]. Cerebrospinal fluid (CSF) rhinorrhea is observed if there is any tear in the meninges, usually accompanied by increased intracranial pressure [1]. CSF rhinorrhea suggests that there is communication between the intracranial subarachnoid space and the sinonasal mucosa, resulting in an increased risk of meningitis [1-2].

Our aim is to underline the high efficiency of the endoscopic surgical treatment and the significance of early diagnosis and effective, definite surgical treatment by presenting an interesting case of a massive, neglected, postoperative meningoencephalocele.

## Case presentation

A 30-year-old man was transferred to the Department of Otolaryngology - Head and Neck Surgery of Evangelismos General Hospital, Athens, Greece, due to a massive meningoencephalocele, resulting in meningitis and coma. His surgical history included right eye mining during infancy due to Coats disease. At the age of 7 years, he developed CSF rhinorrhea, which was attributed to his previous operation. Five years later, he underwent open surgical repair in order to close the deficit and control the CSF leak. Four days postoperatively, the CSF rhinorrhea reappeared. He underwent lumbar diversion twice, one and nine years later respectively, due to persistence of the CSF leak, which resulted in meningitis. Despite efforts, CSF rhinorrhea never ceased, and the patient suffered recurrent episodes of meningitis, with the last one leading to coma and long-lasting admission in the intensive care unit.

**Figure 1:**
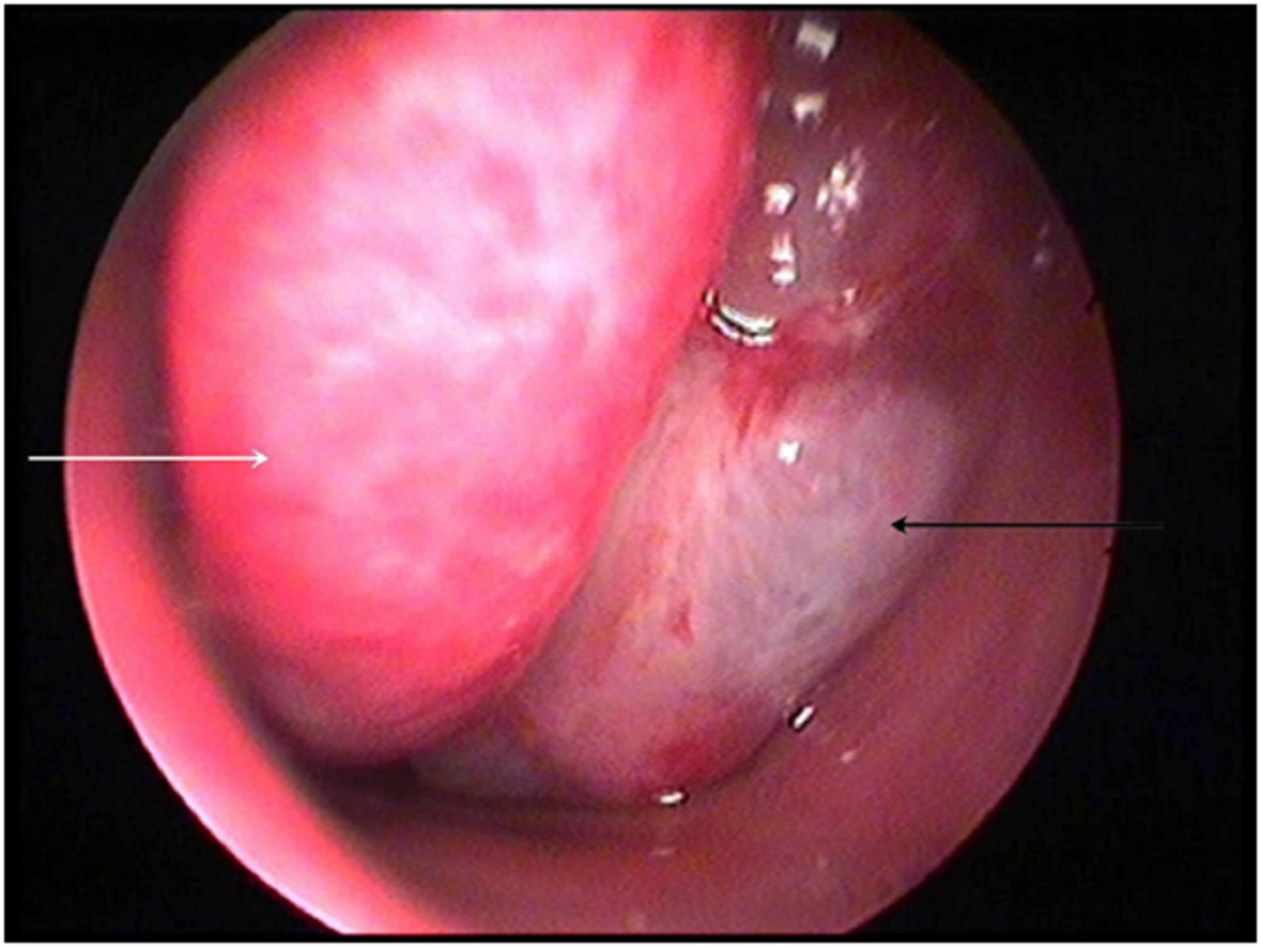
Nasal endoscopy image demonstrating a cystic/polypoid lesion (black arrow) adherent to the middle meatus (white arrow) of the right nasal cavity.

**Figure 2:**
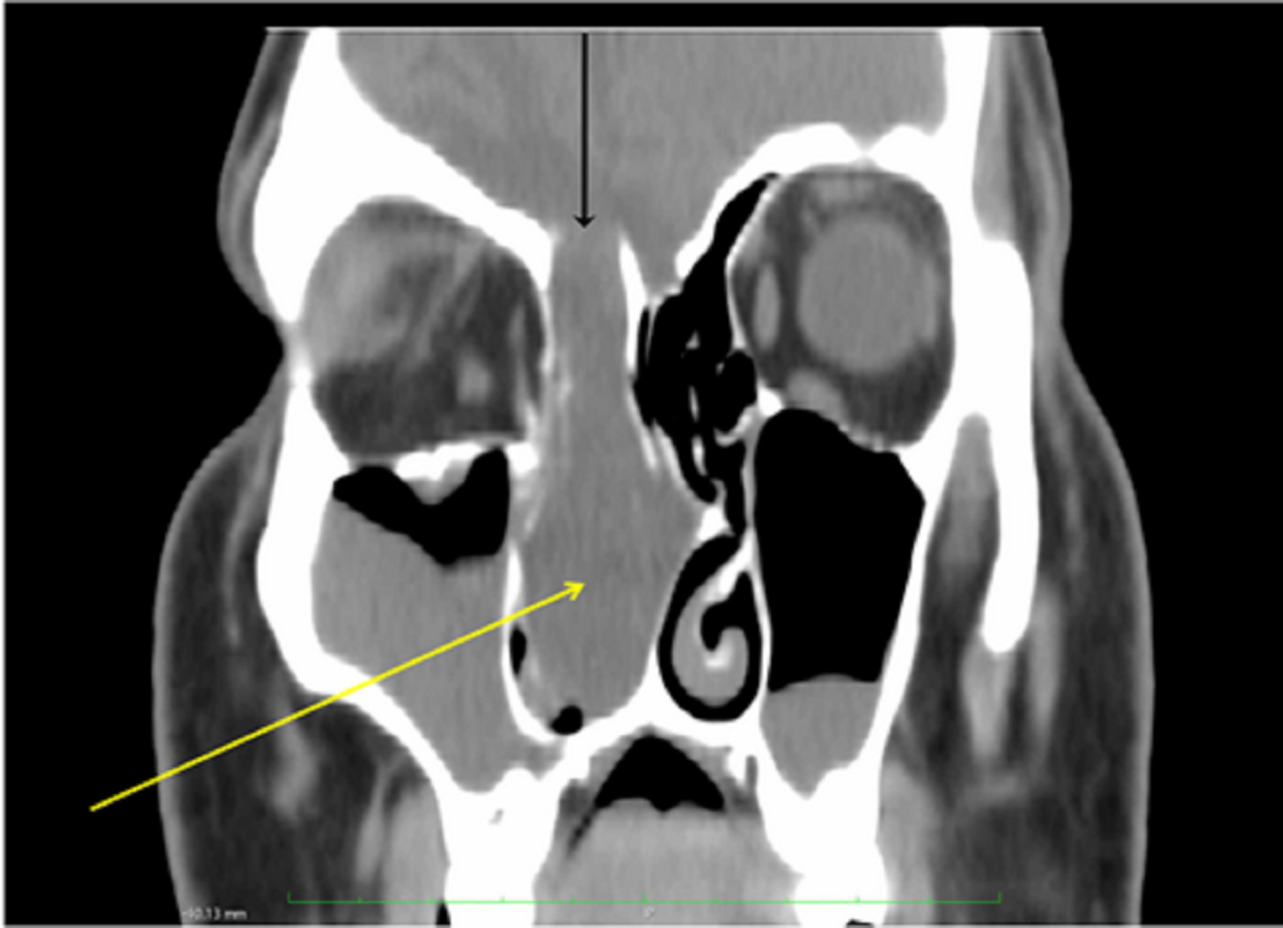
Preoperative craniofacial CT scan (coronal view) revealing a frontal bone gap around the top of the right ethmoid cells (black arrow) and a mass on the roof of the right nasal cavity. The rest of the right nasal cavity was filled with fluid with density similar to CSF (yellow arrow). CSF, cerebrospinal fluid

**Figure 3:**
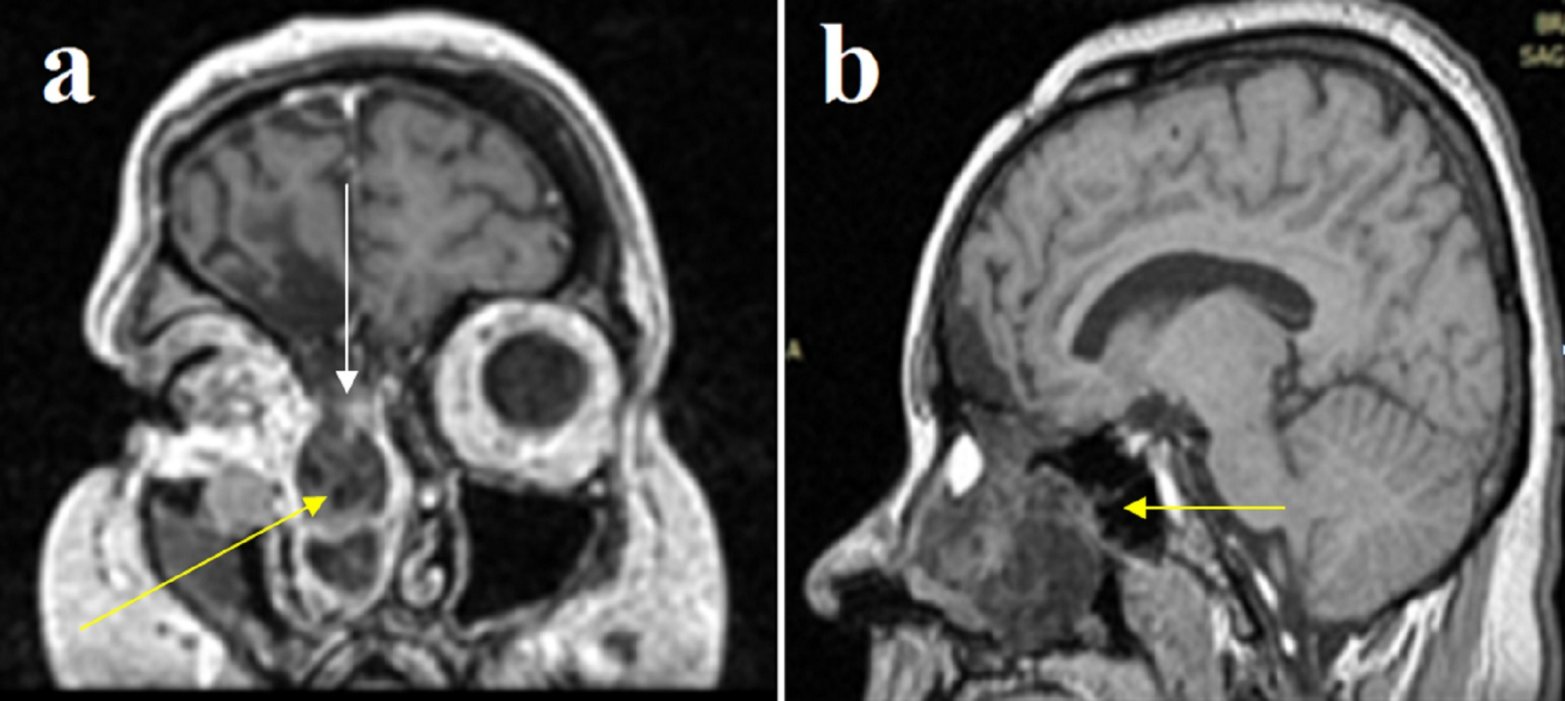
Preoperative MRI brain scan (coronal [a] and sagittal [b] view) demonstrating a large meningoencephalocele extending through a frontal bone gap on the right side (yellow arrows) and a cribriform plate defect with complete opacification of the frontal and anterior ethmoid sinuses (white arrow).

Nasal endoscopy revealed a cystic/polypoid lesion adherent to the middle meatus of the right nasal cavity, causing complete obstruction (Figure 1). Clear fluid leakage was noticed from the area near the neck of the mass. Preoperative cranial computed tomography (CT) scan demonstrated a frontal bone gap around the top of the right ethmoid cells and a soft-tissue sizable (diameter of 1.6 cm) mass on the roof of the right nasal cavity, whereas the rest of the right nasal cavity was filled with fluid with density similar to that of CSF (Figure 2). Magnetic resonance imaging (MRI) of the paranasal sinuses revealed a large meningoencephalocele extending through the right frontal posterior table and a cribriform plate defect with complete opacification of the frontal and anterior ethmoid sinuses (Figure 3).

**Figure 4:**
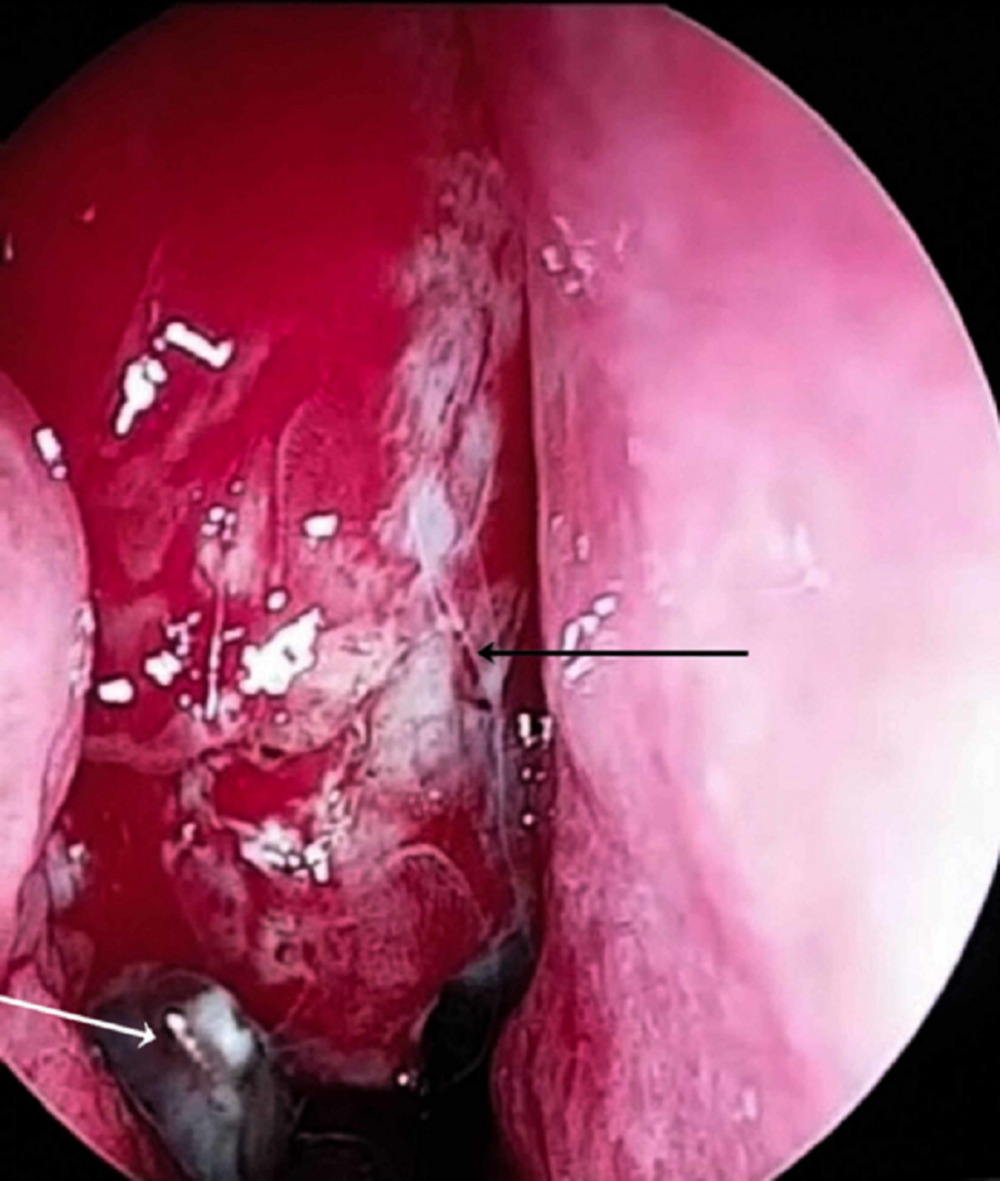
Intraoperative image demonstrating the removal of meningoencephalocele (black arrow) using bipolar diathermy (white arrow).

**Figure 5:**
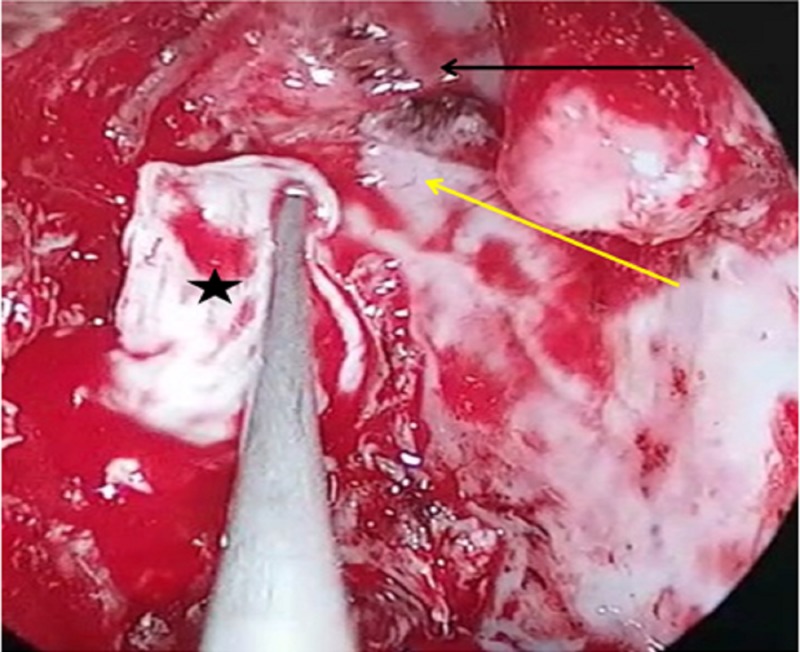
Intraoperative image demonstrating the insertion of the first fascia layer (black star) underlay between the skull base (yellow arrow) and the dura mater (black arrow).

**Figure 6:**
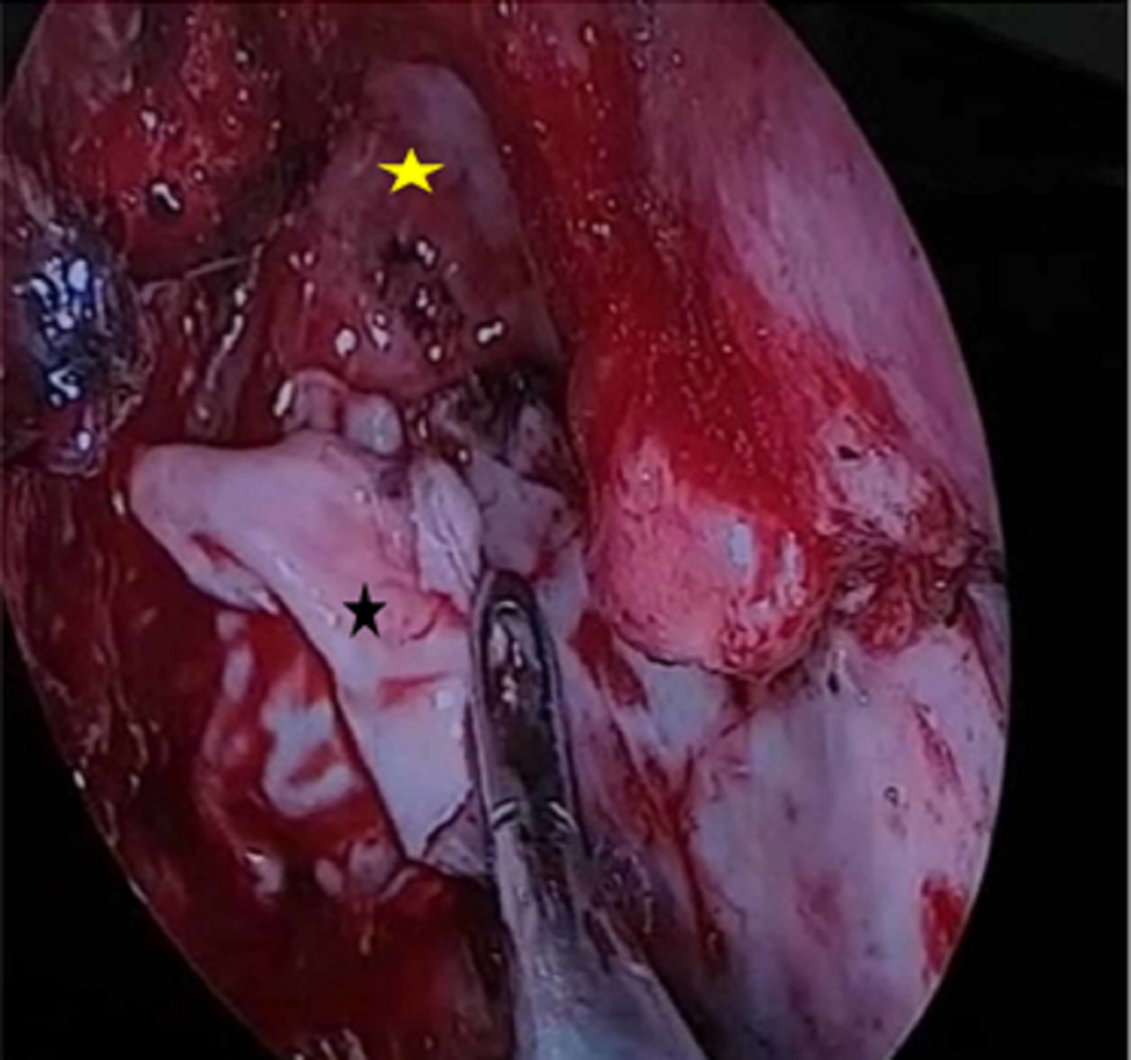
Intraoperative image demonstrating the insertion of the second fascia layer (black star) overlay above the bone-skull base (yellow star).

**Figure 7:**
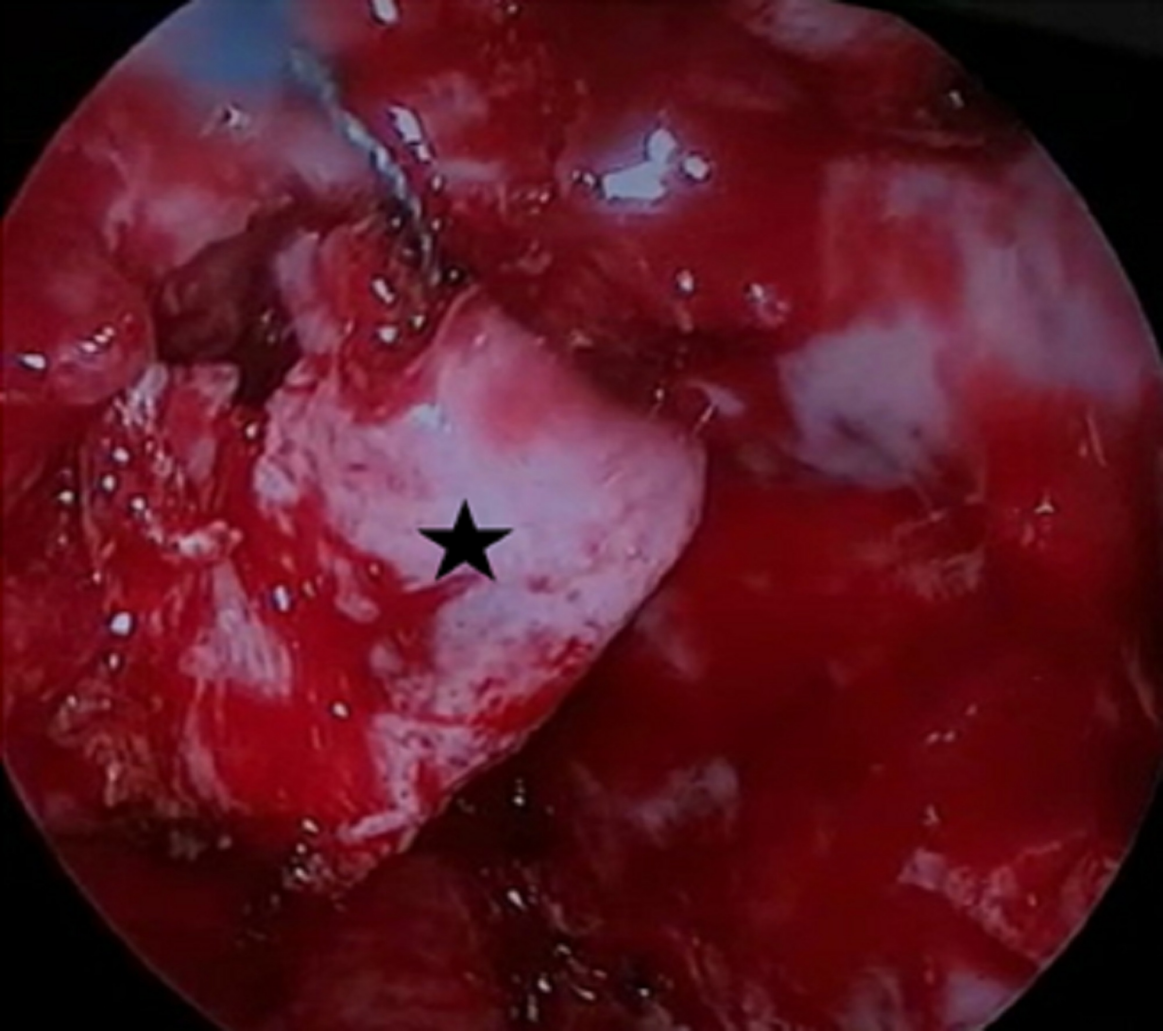
Intraoperative image demonstrating the insertion of vascularized nasoseptal flap (black star) onlay.

The patient underwent extended endoscopic endonasal surgery under general anesthesia in order to resect the right anterior skull-base meningoencephalocele and repair the CSF leak. Intraoperatively, the root of meningocele and the bony defect were recognized using a navigator with real-time MRI fusion. The meningoencephalocele was gradually dissected and completely removed using bipolar diathermy (Figure 4). The skull base, the posterior wall of the maxillary sinus, and the anterior face of the sphenoid sinus were revealed, and the right frontal sinus was drilled using a Draf IIb (endoscopic frontal sinusotomy) surgical approach. The bony defect (width: 1.2 cm; length: 1.9 cm) was then recognized. The mucosa around the bony defect was carefully dissected using bipolar electrocautery, and small unstable pieces of bone were also removed. Afterward, fascia lata was harvested from the thigh in order to begin reconstruction. Two layers of fascia were inserted, the first one between the bone and the dura mater (Figure 5) and the second one above the skull base (Figure 6), followed by insertion of a third layer of vascularized nasoseptal flap onlay (Figure 7). The mucosal flap was placed on the defect, and fibrin glue was sprayed over the edges of the flap using a double-loop catheter. Subsequently, the nasal cavity was packed with hemostatic gauze, Merocel nasal tampon (Medtronic, Minneapolis, MN), and gauzes impregnated with antibiotic ointment.

**Figure 8:**
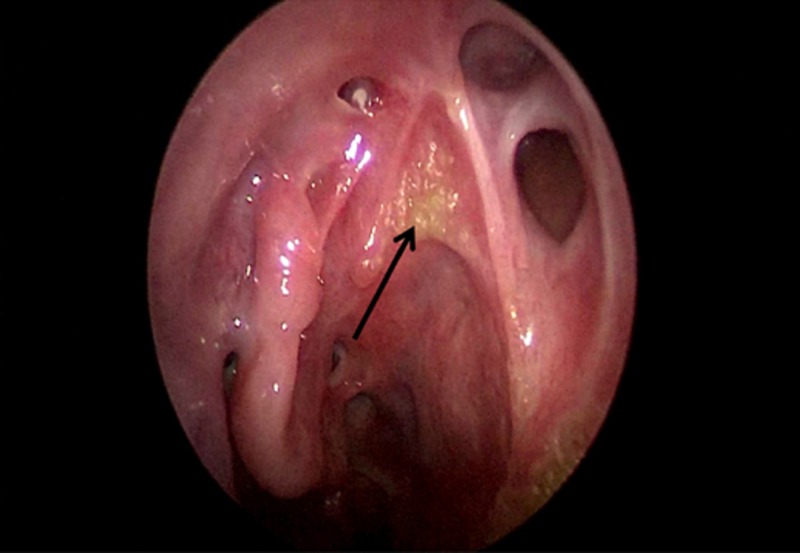
Endoscopic photo demonstrating no recurrence one year postoperatively. Black arrow demonstrates the point where the flaps were placed.

**Figure 9:**
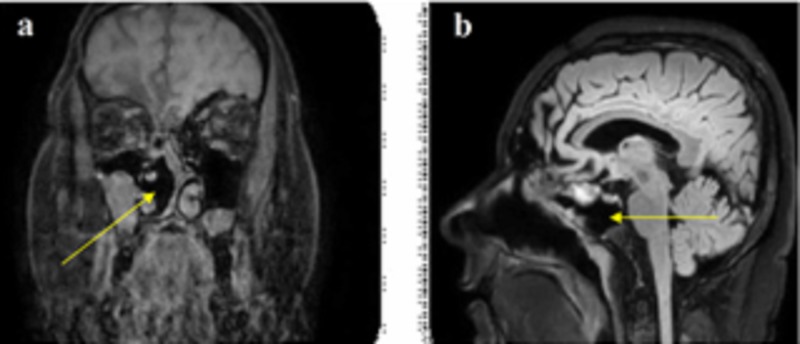
MRI brain scan [coronal (a) and sagittal (b) view] one year postoperatively demonstrating no recurrence of meningoencephalocele into the right nasal cavity (yellow arrow) and no evidence of CSF.

The patient had an uneventful postoperative course in the regular ward and was discharged 48 hours postoperatively. During a follow-up period of 12 months, the patient presented with no CSF rhinorrhea, and subsequent endoscopic and imaging assessment demonstrated no recurrence (Figures 8, 9).

## Discussion

Meningoencephalocele and CSF rhinorrhea can be iatrogenic, congenital, acquired (due to trauma or erosion), or idiopathic in origin [3]. The posterior part of the skull is the most common site of meningoencephalocele in cases in Western Europe and the United States of America, whereas meningoencephalocele of the anterior part of the skull is more often observed in cases in Africa, Russia, and Southeast Asia [3]. Around 90% of the cases are encountered in the median line [4]. Based on the anatomical site of the defect in the cranium, they are classified as transsphenoidal, sphenoethmoidal, spheno-orbital, and transethmoidal [5]. Basal encephaloceles are rare [3].

The main symptom of meningoencephalocele is CSF fluid leak [1-2]. CSF leak has been associated with 10% per year risk of developing meningitis [1]. Other common symptoms of basal meningoencephalocele include seizures, headaches, respiratory problems, and endocrinal abnormalities [1].

Imaging (CT scan, MRI) and nasal endoscopy confirm the diagnosis, evaluate the underlying cause, and localize and characterize the defect site prior to surgical repair [5].

The main targets of meningoencephalocele treatment are to avoid relapse and cease the CSF leak [5]. Conservative treatment of CSF leakage is associated with a 19-29% incidence of meningitis [4-5]. Therefore, surgical excision of the herniated tissue and surgical repair of the deficit is the only viable solution [6-7]. The intracranial approach has a success rate of 70-90%, whereas the transnasal endoscopic technique has a success rate of 80-90% [5].

Endoscopic technique offers reduced postoperative morbidity and reduced length of hospitalization (usually 48 hours), which were confirmed in our case, and avoids the complications of craniotomy, such as anosmia and intense headaches [8]. Endoscopic technique can be applied to either small or large defects [5]. The average calculated risk of recurrence of CSF leak is 21%, with the complication rate increasing with increased duration of uncorrected leak [5]. A second attempt in case of relapse has a success rate of 97%, underlining that a prior failed surgical treatment of defect is not an indication to proceed with diversion or conservative treatments [7-9]. In our case, the patient was subjected to lumbar diversion twice following failed surgical treatment, resulting in recurrent episodes of meningitis and being exposed to a risk of permanent neurological damage and death each time. This condition lasted from his childhood until his late twenties and severely affected the quality of his life, whereas a simple (lasting two hours) operation finally resolved the long-lasting medical condition. While efforts to reduce the pressure of CSF have a therapeutic meaning, they should only be applied after the deficit has been closed and intracranial hypertension has been diagnosed [7,9]. Surgical excision of the herniated tissue and repair of the bony deficit is the only viable therapeutic solution [1,2,5,7,9].

## Conclusions

Endoscopic treatment of meningoencephalocele is a highly successful, minimally invasive treatment option with low morbidity. Preoperative imaging and planning by experienced head and neck surgeons are of paramount importance in order to select the appropriate reconstructive method and to achieve the optimal outcome for the patient. Effective, definite and timely surgical repair is crucial in order to avoid life-threatening complications, improve patient’s and caregiver’s quality of life, and avoid unnecessary health-system costs.
